# Non-pharmacological Perioperative Interventions for the Prevention of Postoperative Delirium in Elderly Patients Undergoing Hip and Knee Arthroplasty Surgery: A Systematic Review

**DOI:** 10.7759/cureus.99475

**Published:** 2025-12-17

**Authors:** Mohamed Zahed, Ziad El Menawy, Mahmoud Elmesalmi, Nour Elnaggar, Farouk Ahmed, Rawad M Azaz, Ahmed Elkilany, Sherif I Elhabbak, Seifeldin H Amer, Manar Adel

**Affiliations:** 1 Orthopaedics, John Radcliffe Hospital, Oxford University Hospitals NHS Trust, Oxford, GBR; 2 Trauma and Orthopaedics, University Hospital of Wales, Cardiff, GBR; 3 Trauma and Orthopaedics, St George's University Hospitals, London, GBR; 4 Faculty of Medicine, Zagazig University, Zagazig, EGY; 5 Emergency Medicine, Queen Alexandra Hospital, Portsmouth, GBR; 6 Trauma and Orthopaedics, London Royal Free NHS Trust, London, GBR; 7 Trauma and Orthopaedics, Worthing Hospital, University Hospitals Sussex NHS Foundation Trust, Worthing, GBR; 8 General Practice, Faculty of Medicine and Surgery, October 6 University, Giza, EGY; 9 Clinical Pharmacy, Tanta University, Gharbia, EGY

**Keywords:** elderly, hip arthroplasty, knee arthroplasty, non-pharmacological interventions, perioperative care, postoperative delirium, systematic review

## Abstract

Postoperative delirium is common in elderly patients after orthopedic surgery. While pharmacological interventions often prove ineffective and are associated with significant adverse effects, non-pharmacological strategies targeting modifiable risk factors show promise. This systematic review evaluates perioperative non-pharmacological interventions for preventing postoperative delirium in elderly adults undergoing total knee or hip arthroplasty.

This systematic review was conducted following PRISMA (Preferred Reporting Items for Systematic Reviews and Meta-Analyses) guidelines. Five electronic databases were searched for randomized controlled trials and observational studies examining non-pharmacological interventions to prevent postoperative delirium in patients aged 60 years or older undergoing total knee or hip arthroplasty. Two independent reviewers performed study selection, data extraction, and quality assessment using the Cochrane Risk of Bias tool (RoB 2) for randomized trials and Risk of Bias in Non-randomized Studies of Interventions (ROBINS-I) for non-randomized studies.

Eleven studies comprising 15,761 patients were included. Postoperative delirium incidence ranged from 0% to 34% across interventions. The fast-track methodology achieved the lowest incidence at 0%, while maintaining 10-20% below baseline showed the highest incidence at 34%. Tourniquet use was associated with higher delirium rates (19.1% vs 9.6%; 15.22% vs 5.43%) and increased pain scores. Comprehensive care protocols reduced delirium incidence (15.9% vs 30.8%) and duration (2.06 vs 3.42 days) compared to conventional care. Fresh frozen plasma transfusion demonstrated a 5.96-fold increased delirium risk. Transcranial direct current stimulation reduced the incidence of delirium from 19.7% to 4.9%. Hospital length of stay ranged from 2.6 days with fast-track methodology to 15 days with suboptimal blood pressure management.

Multimodal non-pharmacological interventions can significantly reduce postoperative delirium in elderly orthopedic surgery patients. Evidence-based strategies include maintaining adequate intraoperative blood pressure, implementing fast-track care pathways, avoiding unnecessary fresh frozen plasma transfusions, and adopting comprehensive care bundles. Standardized delirium prevention protocols should be prioritized in clinical practice to improve outcomes in this vulnerable population.

## Introduction and background

Delirium represents a critical neuropsychiatric syndrome manifesting as acute-onset disturbances in attentional processes and broader cognitive function. The condition frequently presents with fluctuating levels of consciousness, spanning a spectrum from hypoactive states characterized by profound unresponsiveness bordering on stupor to hyperactive presentations [1]. Patients commonly exhibit distressing psychotic phenomena, including hallucinatory experiences across sensory modalities, delusional ideation, and affective dysregulation [2].

The condition presents in three motor subtypes: hyperactive (agitation and restlessness), hypoactive (lethargy and decreased responsiveness), and mixed (alternating features), with hypoactive delirium being most common yet frequently underrecognized. Core diagnostic features include acute onset, inattention, disorganized thinking, and altered consciousness, while perceptual disturbances, sleep-wake disruption, and emotional dysregulation may also occur [3]. Postoperative delirium (POD) constitutes the most prevalent surgical complication and represents a substantial burden on population health outcomes [4]. Over half of surgical patients who manifest delirium subsequently develop cognitive deficits that may persist for up to 12 months following the operative procedure [5].

Total joint arthroplasty (TJA), including total hip arthroplasty (THA) and total knee arthroplasty (TKA), is among the most effective surgical interventions for managing advanced degenerative joint disease and osteoarthritis [6,7]. These procedures significantly reduce pain, restore functional capacity, and substantially improve patients' quality of life. The utilization rates of THA and TKA have increased dramatically and are projected to continue rising in both the United States and Europe, positioning these procedures as an increasingly significant public health concern [8,9]. Due to the surgical complexity inherent to TJA, patients undergoing these procedures face a particularly elevated risk of developing postoperative complications, with POD being one of the most prevalent adverse outcomes, especially among elderly populations. POD following TJA results in serious consequences, including delayed recovery, extended hospital stays, increased healthcare costs, and elevated mortality risk [10,11]. The reported incidence of POD is notably high in TKA patients, with similarly elevated rates observed following THA procedures, making delirium prevention and management a critical priority in orthopedic surgical care [12,13].

Conventional delirium management focuses on treating underlying medical etiologies, though pharmacologic agents are commonly employed to address associated symptoms such as agitation and hallucinations. However, evidence supporting the efficacy and risk-benefit profile of these medications, particularly antipsychotics, in hospitalized patients remains uncertain [14]. Given these limitations and the multifactorial nature of POD, particularly in the TJA population, there has been increasing interest in non-pharmacological interventions as safer alternatives or adjuncts to traditional management strategies [15].

Non-pharmacological interventions target modifiable risk factors and aim to optimize the perioperative environment without the adverse effects associated with pharmacotherapy. These multidisciplinary approaches encompass multicomponent strategies including early mobilization, cognitive stimulation, sleep hygiene optimization, adequate pain management, nutritional support, hydration protocols, sensory aids (visual and auditory), environmental modifications, reorientation techniques, familiarity enhancement, effective communication strategies, and minimizing ecological disruptions [16]. Evidence from various surgical populations demonstrates that bundled non-pharmacological interventions can significantly reduce delirium incidence. However, in patients undergoing TJA, the individual effectiveness of specific intervention components and their impact on postoperative outcomes, including delirium prevention, morbidity, mortality, and post-discharge quality of life, remain poorly understood [17].

To address this knowledge gap, this systematic review aimed to evaluate the effectiveness of perioperative non-pharmacological interventions in reducing the incidence, duration, and severity of POD in elderly patients undergoing elective or emergency knee or hip arthroplasty.

## Review

Methods

The study design was conducted in accordance with the Cochrane Handbook for Systematic Reviews of Interventions [18]. The manuscript was prepared following the Preferred Reporting Items for Systematic Reviews and Meta-Analyses (PRISMA) guidelines [19].

Search Strategy

A comprehensive systematic literature search was performed across multiple electronic databases to identify studies examining the effectiveness of non-pharmacological interventions in preventing or managing postoperative delirium following knee or hip arthroplasty. The databases included PubMed, Web of Science (WOS), Scopus, Embase, and the Cochrane Library. The search strategy employed a combination of controlled vocabulary (MeSH terms) and free-text keywords related to "postoperative delirium," "non-pharmacological interventions," "total knee arthroplasty," and "total hip arthroplasty." Database-specific search terminology is presented in the Appendices.

Study Selection

All records identified through database searches were imported into EndNote software version X9 (Clarivate Analytics, Philadelphia, Pennsylvania). Duplicate records were eliminated, and the remaining citations underwent a two-tier screening procedure, consisting of title and abstract review followed by full-text evaluation. Two independent investigators screened titles and abstracts during the preliminary phase using established eligibility criteria. Any disagreements were addressed through collaborative discussion until consensus was reached.

Inclusion Criteria

Studies were included if they met the following criteria: participants were elderly patients (≥60 years) undergoing elective or emergency TKA or THA; the intervention involved perioperative non-pharmacological interventions; the comparator was standard care, usual care, or alternative non-pharmacological intervention strategies; outcomes included the incidence of postoperative delirium as the primary outcome, with secondary outcomes including delirium severity, delirium duration, hospital length of stay, in-hospital mortality, ICU admission, mechanical ventilation, falls, pressure ulcers, urinary tract infections, pneumonia, use of physical restraints, unplanned surgery, and rescue medication use; the study design comprised randomized controlled trials (RCTs), non-RCTs, quasi-experimental studies, and observational studies, including cohort and case-control studies; and studies were full-text, peer-reviewed articles published in English.

Exclusion Criteria

Studies were excluded if they were animal studies or in vitro research; were published in non-English languages; focused exclusively on pharmacological interventions for delirium prevention or management; did not involve knee or hip arthroplasty procedures; did not report postoperative delirium as an outcome; or were conference abstracts, editorials, letters, commentaries, case reports, or review articles without original data.

Data Extraction

Data extraction was independently conducted by two reviewers using a pre-piloted systematic extraction template. Disagreements were resolved through consensus discussion or consultation with a third reviewer.

Risk of Bias Assessment

The risk of bias for RCTs was assessed independently by two reviewers using the Revised Cochrane Risk of Bias tool for randomized trials (RoB 2) [20]. The RoB 2 tool evaluates study quality across five domains: bias arising from the randomization process, bias due to deviations from intended interventions, bias due to missing outcome data, bias in measurement of the outcome, and bias in selection of the reported result. For non-randomized studies and cohort studies, the Risk of Bias in Non-randomized Studies of Interventions (ROBINS-I) tool was employed [21]. ROBINS-I assesses bias across seven domains: bias due to confounding, bias in participant selection, bias in intervention classification, bias due to deviations from intended interventions, bias due to missing data, bias in outcome measurement, and bias in the selection of the reported result. Each study was systematically evaluated for potential sources of bias according to the respective tool’s criteria. Any disagreements between reviewers regarding bias assessment were resolved through collaborative discussion or consultation with a third reviewer when consensus could not be reached. The overall risk of bias for each study was categorized as low risk, some concerns, or high risk for RCTs using RoB 2, and as low, moderate, or serious risk for non-randomized studies using ROBINS-I.

Results

Study Selection

**Figure 1:**
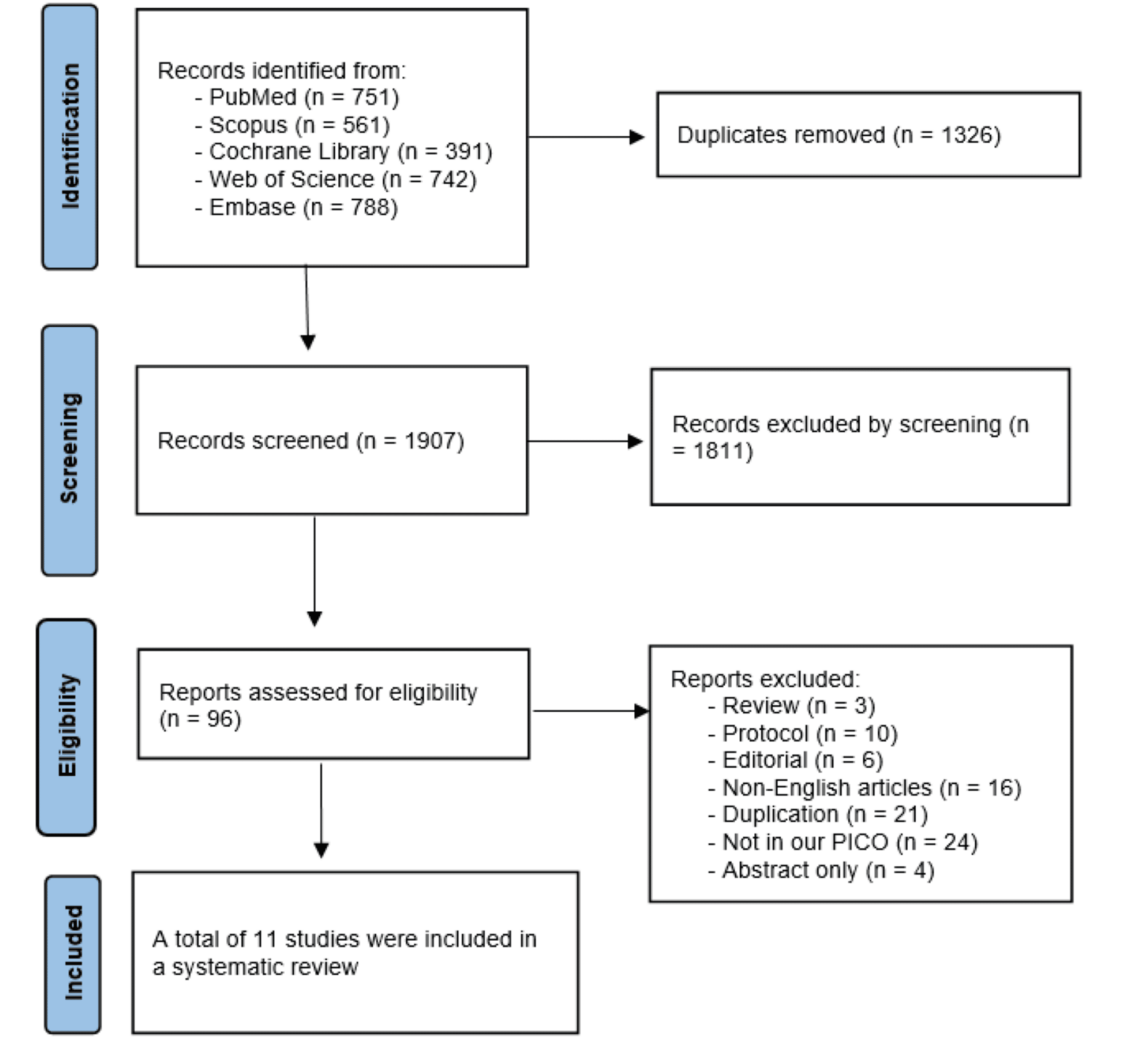
PRISMA flow diagram References: [22-32]. PRISMA: Preferred Reporting Items for Systematic Reviews and Meta-Analyses.

After searching five databases, we collected 3233 records. Removing 1,326 duplicates yielded 1,907 unique records. We then screened the titles and abstracts, excluding 1811 entries. We retrieved the full text of the remaining 96 records and evaluated them against our eligibility criteria. Finally, 11 studies were included in our systematic review [22-32]. The flow diagram illustrating the study selection is shown in Figure 1.

Baseline and Summary of the Included Studies

A total of 11 studies comprising 15,761 patients were included. Patient ages across the included studies ranged from 65.8 to 82.5 years, with most studies focusing on elderly populations. The included studies were conducted across several countries, with China emerging as the predominant contributor (nine studies), followed by Denmark (two studies), South Korea, and Taiwan, collectively investigating various interventions for POD in older adults undergoing orthopedic surgery. The majority of studies employed RCTs or cohort-based designs, with sample sizes ranging from 128 to 6,737 patients. A diverse array of interventions was examined, including tourniquet application, blood transfusion strategies (restrictive versus liberal, autologous versus allogeneic), fast-track care methodologies, transcranial direct current stimulation (tDCS), and perioperative blood pressure management protocols. Baseline cognitive function was assessed using Mini-Mental State Examination (MMSE) scores in several studies, with mean scores generally indicating preserved cognition (≥20). It is important to note that comorbidity data, including hypertension, diabetes, and cardiovascular disease, were not consistently reported across studies, potentially introducing variability in how baseline health status was characterized. Comprehensive baseline and summary characteristics of the included studies are detailed in Tables 1, 2.

**Table 1:** Summary of the included studies References: [22-32]. NCT: National Clinical Trial, POD: postoperative delirium, DSM: Diagnostic and Statistical Manual of Mental Disorders, TKA: total knee arthroplasty, THA: total hip arthroplasty, TJA: total joint arthroplasty, ASA: American Society of Anesthesiologists, MMSE: Mini-Mental State Examination.

Study ID	Trial registry (NCT)	Total patients	Country	Follow-up duration	Primary outcomes	Inclusion criteria
Chen 2025 [22]	ChiCTR2400085505	313	China	7 Days postoperative	Incidence of POD within the postoperative 7 days	≥60 years old, ASA I-III, single TKA under general anesthesia
Fan 2014 [23]	ChiCTR81300946	186	China	7 Days postoperative	Incidence of POD during the first 7 postoperative days	≥60 years, ASA I-III, elective unilateral total hip replacement under spinal anesthesia
Jing 2022 [25]	ChiCTR2200055446	128	China	Postoperative period until discharge	Rate of perioperative delirium	≥60 years, isolated hip fracture
Kim 2024 [26]	NA	6737	Korea	Within 2 days post-op	Delirium within 2 days post-operative	Adults ≥18 years, elective TKA/THA
Krenk 2012 [27]	NCT01103752	225	Denemark	Mean of 12 days	Postoperative delirium (DSM-IV criteria)	Age ≥60, ASA I-IV, MMSE ≥24, fluent Danish, no recent anesthesia
Liang 2021 [28]	NA	140	Taiwan	12 Months	cognitive function	Patients aged 60 years and older scheduled for elective orthopedic surgery
OuYang 2023 [29]	NA	1143	China	During hospital stay	Postoperative delirium	Patients ≥ 65 years undergoing their first TJA with normal preoperative cognitive function; patients suffering from hip or knee osteoarthritis and femoral head necrosis
Petersen 2017 [30]	NCT01515670	6331	Denmark	Up to 4 days postoperatively	Delirium after fast-track hip and knee arthroplasty	Patients undergoing primary TJA, aged more than 70 years, with normal preoperative function
Ran 2022 [31]	ChiCTR2100045711	184	China	3 Days after surgery	Incidence of POD within 72 h after surgery	Patients undergoing TKA with general anesthesia, aged more than 65 years, with ASA classification I-III, and no history of drug allergy
Tao 2023 [32]	ChiCTR2200057024	122	China	3 Days postoperatively	Incidence of POD during the first 3 postoperative days	Age ≥65 years, ASA ≤3, scheduled for THA or TKA, and MMSE score ≥15
Xu 2020 [24]	ChiCTR1900022411	150	China	3 Days postoperatively	Incidence of POD during the first 3 postoperative days	Age 65-80 years, ASA II-III, NYHA class II-III, undergoing elective hip replacement, and MMSE score above the cutoff for education level.

**Table 2:** Baseline characteristics of the included studies References: [22-32]. BMI: body mass index, ASA: American Society of Anesthesiologists, MMSE: Mini-Mental State Examination, SD: standard deviation, MI: myocardial infarction.

Study ID	Study arms, (n)	Age (years, mean ± SD)	Gender (male, %)	BMI (kg/m², mean ± SD)	ASA classification (I/II/III) - n(%)	MMSE score (cognitive score)(mean ± SD)	Fracture type/location - n(%)	Hypertension - n(%)	Diabetes- n(%)	Cardiovascular disease - n(%)
Chen 2025 [22]	Tourniquet (n = 157)	69.2 ± 5.5	29.90%	26.1 ± 3.2	I: 0.6%, II: 68.8%, III: 30.6%	22.0 (20.0-24.0)	NA	61.10%	18.50%	10.20%
	No tourniquet (n = 156)	69.3 ± 6.3	26.90%	25.8 ± 2.9	I: 0.6%, II: 65.4%, III: 34%	22.0 (20.0-24.0)	NA	63.50%	16.70%	14.70%
Fan 2014 [23]	Restrictive transfusion (n = 94)	73 ± 7	31.90%	59 ± 10 kg (weight)	I: 6%, II: 75%, III: 13%.	28.3 ± 1.7	Hip fracture (total hip replacement)	55.30%	13.80%	9.60%
	Liberal transfusion (n = 92)	75 ± 6	34.80%	58 ± 11 kg (weight)	I: 5%, II: 73%, III: 14%	28.3 ± 1.7		62.00%	16.30%	10.90%
Jing 2022 [25]	Comprehensive care (n = 63)	75.3 ± 2.2	44.40%	NA	NA	NA	Femoral head: 38.1%, Intertrochanteric: 52.4%, Subtrochanteric: 9.5%	33.30%	NA	22.20%
	Conventional care (n = 65)	73.5 ± 6.1	47.70%	NA	NA	NA	Femoral head: 41.5%, Intertrochanteric: 53.9%, Subtrochanteric: 4.6%	27.60%	NA	26.10%
Kim 2024 [26]	Blood transfusion (n = 868)	63.59 ± 14.68	26.15%	NA	I: 19%, II: 59%, III: 22%	NA	Knee:61%, Hip:39%	NA	15.67%	21.61%
	Non-blood transfusion (n = 5869)	66.52 ± 12.16	27.06%	NA	I: 15%, II: 70%, III: 14%.	NA		NA	23.50%	18.98%
Krenk 2012 [27]	Fast-track methodology (n = 225)	69.4 (60-86)	21.40%	27.3	I:31%, II:64%, III:6%, IV:0%	28.6 (24-30)	NA	56.00%	8.00%	22.63%
Liang 2021 [28]	Modified Hospital Elder Life Program (n = 81)	70.7±5.4	18.50%	28.5±5.0	NA	20.3±4.9	Knee 100%	56%	22%	10%
	Usual care (n = 59)	71.9 ± 6.0	25.40%	28.5±4.0	NA	21.5±3.7		58%	22%	10%
OuYang 2023 [29]	Autologous blood transfusion (n = 401)	68 (66-71)	73.10%	26.6 (24.5-29.0	I-II: 375 (93.5%), III-IV: 26 (6.5%)	NA	TKA: (82.0%), THA: (18.0%)	52.90%	18.70%	Heart failure: 1.2%, MI: 0.7%, Coronary heart disease: 5.2%
	Allogeneic blood transfusion (n = 742)	70 ± 5.19	73.70%	26.43 ± 3.64	I-II: 647 (87.2%), III-IV: 95 (12.8%)	NA	TKA:(76.7%), THA: (23.3%)	56.50%	20.40%	Heart failure: 1.3%, MI: 3.0%, Coronary heart disease: 10.4%
Petersen 2017 [30]	fast-track methodology with LOS > 4 days (n = 789)	76.68 ± 0.15	37.30%	NA	NA	NA	NA	NA	NA	NA
Ran 2022 [31]	Tourniquet (n = 92)	70.88 ± 5.24	25.00%	25.37 ± 3.55	II: 40 (43.5%), III: 52 (56.5%)	28.25 ± 0.56	TKA only (100%)	35.90%	10.90%	5.40%
	No tourniquet (n = 92)	70.86 ± 5.1	21.70%	25.21 ± 3.76	II: 42 (45.7%), III: 50 (54.3%)	28.11 ± 0.56		28.30%	16.30%	6.50%
Tao 2023 [32]	Active-tDCS (n = 61)	70.3 ± 6.83	42.60%	24.8 ± 3.2	ASA II: 31 (50.8%), ASA III: 30 (49.2%)	29 (28.0-29.0)	TKA: (63.9%), THA (36.1%)	22%	11.50%	8.20%
	Sham-tDCS (n = 61)	71 ± 5.31	26.20%	24.6 ± 3.8	ASA II: 24 (39.3%), ASA III: 37 (60.7%)	29 (28.0-29.0)	TKA: (60.7%) THA: (39.3%)	24%	24.60%	21.30%
Xu 2020 [24]	BP 10% to 20% below the baseline (D) (n = 50)	69 ± 8	40%	25 ± 4	ASA II : 38(76%),ASA III : 12(24%)	NA	Hip 100%	54%	16%	NA
	BP from baseline to 10% below the baseline (M) (n = 50)	69 ± 8	38%	24 ± 4	ASA II : 36(72%),ASA III : 14(28%)	NA	Hip 100%	52%	14%	NA
	BP from baseline to 10% above the baseline (H) (n = 50)	68 ± 6	42%	23 ± 3	ASA II : 38(76%),ASA III : 12(24%)	NA	Hip100%	46%	19%	NA

Quality Assessment

**Figure 2:**
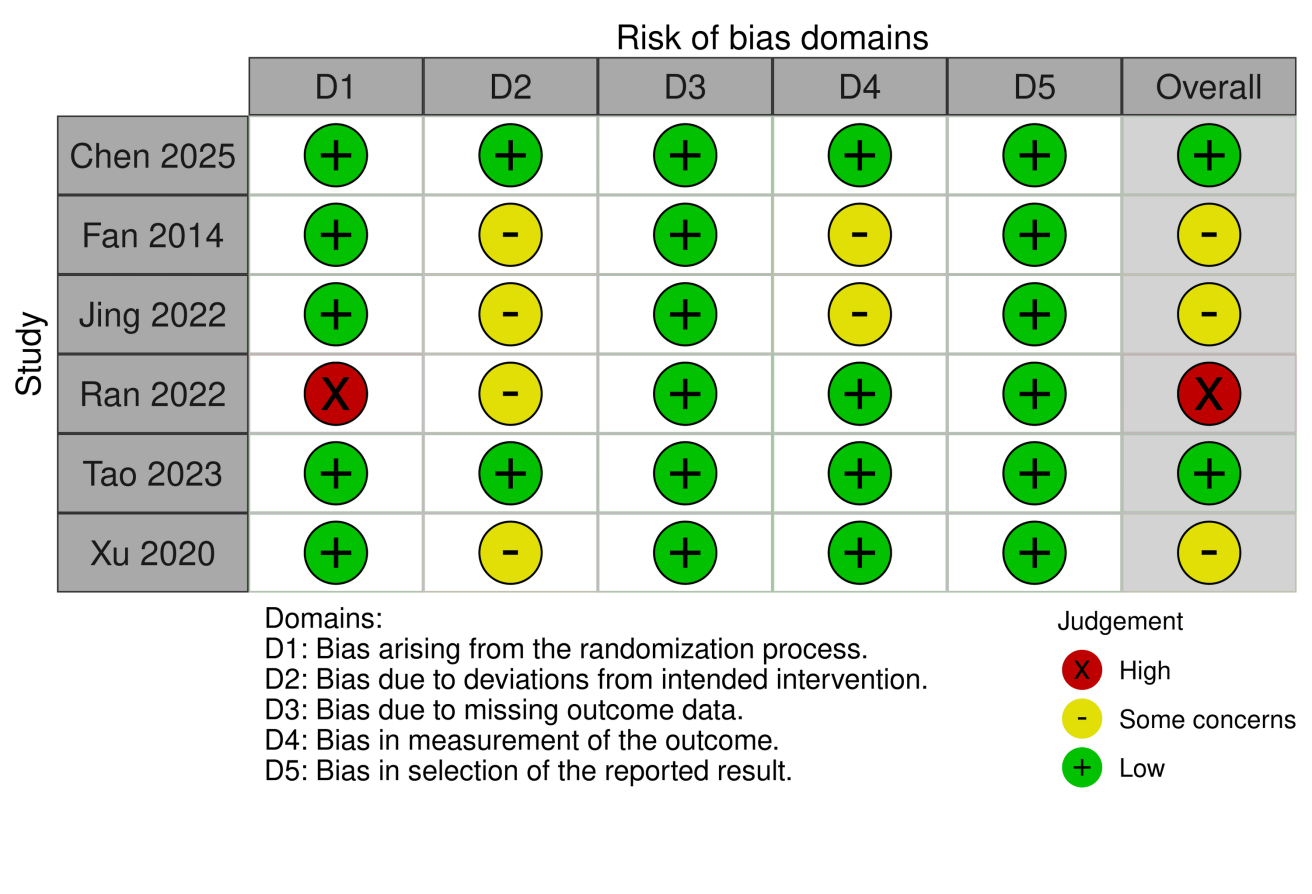
Risk of Bias 2 References: [22-25,31,32].

**Figure 3:**
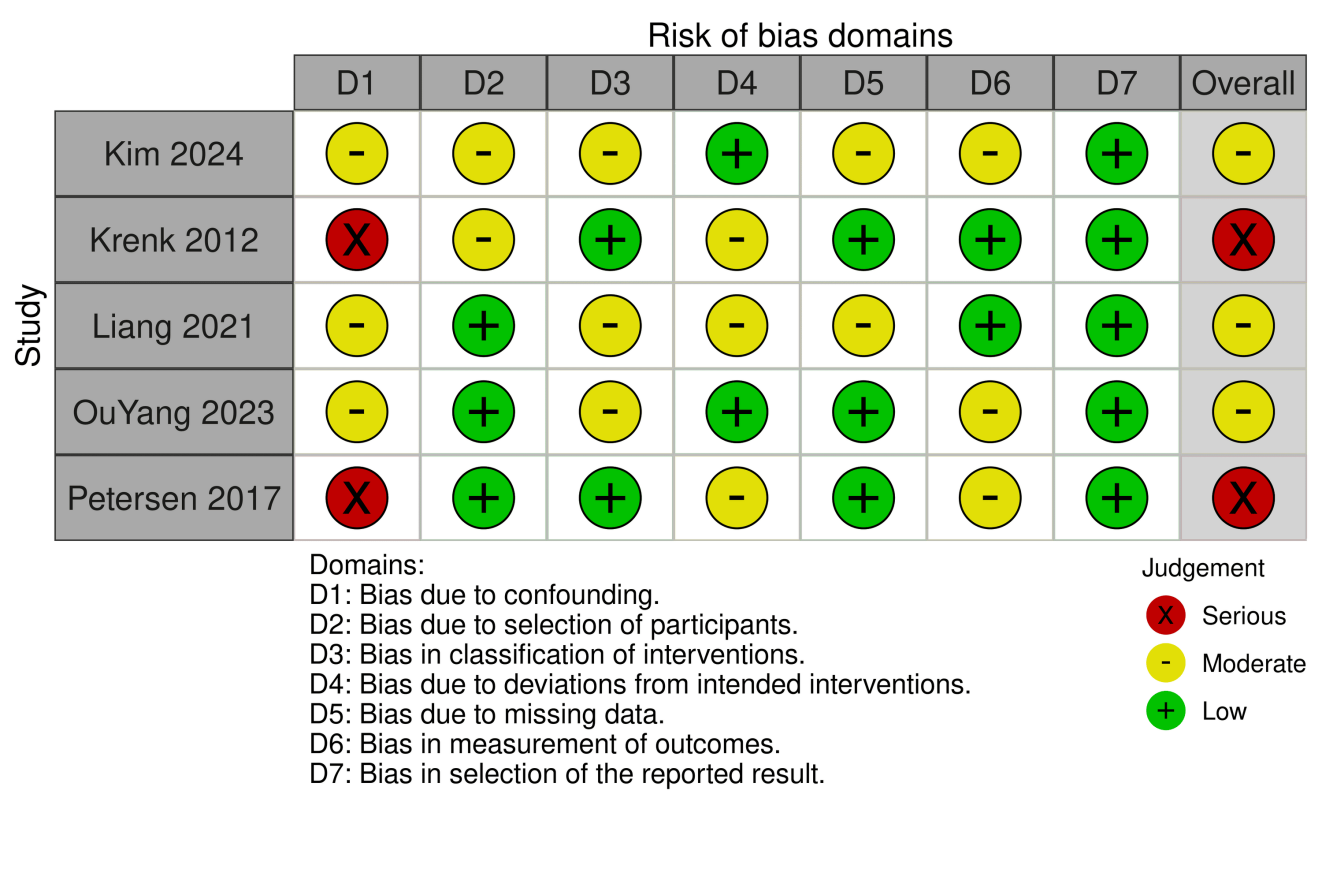
Risk of Bias in Non-randomized Studies References: [26-30].

Among the six RCTs assessed using ROB2, two studies demonstrated an overall low risk of bias. Three studies were rated as having some concerns overall, primarily due to deviations from the intended intervention (D2) and bias in the measurement of the outcome (D4). One study (Ran 2022 [31]) was judged to have a high overall risk of bias, attributed to concerns in the randomization process (D1) and deviations from the intended intervention (D2). Of the five non-randomized studies evaluated with ROBINS-I, two studies exhibited a serious overall risk of bias, mainly driven by confounding bias (D1). In comparison, three studies demonstrated a moderate overall risk of bias. Common concerns across the non-randomized studies included bias due to confounding (D1), classification of interventions (D3), missing data (D5), and measurement of outcomes (D6). Overall, the quality of evidence was variable, with approximately half of the included studies demonstrating a low to moderate risk of bias. All data are presented in Figures 2, 3.

Study outcomes

Postoperative Delirium Incidence

Postoperative delirium incidence ranged from 0% to 34% across interventions. The highest rate was observed in Xu et al. (2020) among patients with blood pressure maintained 10% to 20% below baseline (34%), followed by Jing et al. (2022) conventional care (30.8%), Fan et al. (2014) liberal transfusion (23.9%), and restrictive transfusion (21.3%). The lowest incidence was reported in Krenk et al. (2012) fast-track methodology (0%), followed by OuYang et al. (2023) autologous blood transfusion (2%), Kim et al. (2024) non-blood transfusion (2.35%), and Liang et al. (2021) modified Hospital Elder Life Program (2.5%).

Tourniquet use was associated with higher delirium rates. Chen et al. (2025) reported 19.1% with tourniquet use versus 9.6% without tourniquet, and Ran et al. (2022) observed 15.22% versus 5.43%. In Chen et al. (2025), hypoactive delirium predominated in both groups (tourniquet: 18 cases; no tourniquet: nine cases), with fewer hyperactive (seven versus four) and mixed-type (five versus two) cases [22]. Tao et al. (2023) reported lower delirium incidence with active tDCS (4.9%) compared with sham tDCS (19.7%). The hypoactive subtype predominated in the sham group (six cases), while the active group had mainly mixed-type (two cases) and hypoactive (one case) delirium [32].

Blood pressure management in Xu et al. (2020) showed a delirium incidence of 34% at 10% to 20% below baseline, 24% at baseline to 10% below, and 6% at baseline to 10% above [24]. Petersen et al. (2017) found a 5.45% delirium incidence in fast-track patients with hospital stays exceeding four days. Liang et al. (2021) reported 5.1% in usual care versus 2.5% in the modified Hospital Elder Life Program. OuYang et al. (2023) documented a delirium incidence of 6.6% with allogeneic blood transfusion versus 2% with autologous blood transfusion [29].

Delirium Severity Scores

The highest delirium severity score was observed in the Tao et al. (2023) active-tDCS group, which reached 27, followed by the sham-tDCS group, which was 25 [32]. Ran et al. (2022) documented mean CAM-ICU scores of 14.24 (SD 1.67) with a tourniquet and 13.82 (SD 1.05) without a tourniquet. The lowest scores were in Jing et al. (2022), using the DRS-R-98, with comprehensive care 7.1 (SD 2.7) versus conventional care 11.2 (SD 3.0) [25]. Chen et al. (2025) reported comparable scores: tourniquet 18.0 (SD 2.99) versus no tourniquet 18.3 (SD 2.24) [22].

Duration of Delirium

The most extended delirium duration was in Jing et al. (2022), conventional care at 3.42 days (SD 1.57) versus comprehensive care at 2.06 days (SD 1.03) [25]. The shortest duration was in Chen et al. (2025) at 1.67 days (SD 0.75) for both groups [22]. Tao et al. (2023) reported 2 days for both groups [32].

Hemoglobin Changes

The greatest hemoglobin reduction was in Fan et al. (2014), restrictive transfusion at 38.3 g/L (SD 12.6) versus liberal transfusion at 19.3 g/L (SD 13.12). The most minor changes were in the Ran et al. (2022) tourniquet group at 7.02 g/L (SD 20.56) versus 14.81 g/L (SD 18.4) without a tourniquet. Chen et al. (2025) reported 15.67 g/L (SD 8.23) with a tourniquet and 17.0 g/L (SD 7.48) without a tourniquet at 1 day postoperatively [22].

Blood Loss

Total blood loss ranged from 296 mL to 938 mL. Kim et al. (2024) reported the highest blood loss in the transfusion group at 938 mL (SD 881) versus 296 mL (SD 421) in the non-transfusion group [26]. Chen et al. (2025) documented 553.9 mL (SD 304) with tourniquet use versus 600.37 mL (SD 282.47) without a tourniquet. Fan et al. (2014) showed similar blood losses between groups, with 481 mL (SD 176) in the restrictive transfusion group versus 489 mL (SD 213) in the liberal transfusion group [22,23]. OuYang et al. (2023) reported 266.67 mL (SD 148.8) with autologous transfusion versus 333.33 mL (SD 222.83) with allogeneic transfusion [29]. Xu et al. (2020) demonstrated consistent total blood loss across blood pressure groups, with values of 175 mL (SD 80), 174 mL (SD 116), and 173 mL (SD 95) [24]. Regarding intraoperative blood loss, Ran et al. (2022) reported lower values with tourniquet use at 46.67 mL (SD 15) versus 83.33 mL (SD 22.59) without a tourniquet. Tao et al. (2023) recorded 95 mL (SD 64.54) with active tDCS versus 108.33 mL (SD 94.9) with sham tDCS [32].

Length of Hospital Stay

Hospital stay ranged from 2.6 to 15 days. The shortest stay was reported by Krenk et al. (2012) with the fast-track methodology at 2.6 days (SD 1.17). The longest stay was reported by Xu et al. (2020) at 15 days (SD 5) for blood pressure maintained 10% to 20% below baseline and baseline to 10% below, versus 12 days (SD 4) for baseline to 10% above [24]. Jing et al. (2022) reported a median hospital stay of 14.2 days (SD 2.2) for conventional care versus 11.3 days (SD 2.5) for comprehensive care. Petersen et al. (2017) reported a mean stay of 10.3 days (SD 5.37) in patients with prolonged hospitalization and delirium. Chen et al. (2025) documented a length of stay of 4.27 days (SD 0.86) with tourniquet use versus 3.50 days (SD 0.55) without a tourniquet [22]. Liang et al. (2021) reported similar lengths of stay, with 5.4 days (SD 2.3) in the modified Hospital Elder Life Program group versus 5.2 days (SD 1.7) in the usual care group. Fan et al. (2014) reported 8.8 days (SD 2.7) in the restrictive transfusion group versus 9.3 days (SD 3.9) in the liberal transfusion group [23]. Tao et al. (2023) showed comparable hospital stays, with 9.83 days (SD 2.66) in the active tDCS group versus 9.67 days (SD 3.04) in the sham tDCS group [32].

Pain Outcomes

Ran et al. (2022) documented significantly higher pain with tourniquet use, with a VAS score of 4.43 (SD 0.52) versus 3.38 (SD 0.69) without a tourniquet, and 35.87% versus 13.04% of patients experiencing severe pain. Xu et al. (2020) found no significant differences in VAS scores across blood pressure groups, reporting values of 2.2 (SD 0.8), 2.4 (SD 0.9), and 2.3 (SD 0.9) at one day postoperatively [24,31].

Postoperative Complications

Chen et al. (2025) reported the highest complication burden. Sixty-one patients with tourniquet use and 45 without tourniquet experienced deep vein thrombosis, acute kidney injury, and pneumonia, with no significant differences between groups [22]. OuYang et al. (2023) found that seven patients receiving autologous transfusion and 39 receiving allogeneic transfusion developed deep vein thrombosis and/or required secondary surgery. Lung infection rates were significantly higher with allogeneic transfusion, without a direct correlation [29]. Fan et al. (2014) reported that 13 patients in the restrictive transfusion group and 18 in the liberal transfusion group experienced complications. However, IL-8 levels were significantly higher in the liberal transfusion group at four hours postoperatively, while TNF-α, IL-1β, and IL-6 showed no significant differences [23]. Krenk et al. (2012) documented seven patients with complications, three of whom required reoperation [27].

Blood Transfusion Components and Delirium Risk

Kim et al. (2024) found that fresh frozen plasma transfusion was associated with a 5.96-fold higher incidence of delirium compared with no transfusion (adjusted odds ratio: 5.96, 95% CI: 2.72-13.04; p < 0.001). Other blood components, including red blood cells, platelets, and cryoprecipitate, showed no significant association with delirium [26].

Functional and Cognitive Outcomes

Liang et al. (2021) assessed outcomes at one month. tMMSE scores were comparable, with 71 patients in the modified Hospital Elder Life Program scoring 20.8 (SD 4.2) versus 52 patients in the usual care group scoring 21.1 (SD 4.1). ADL scores were also similar, with 78 patients in the modified Hospital Elder Life Program scoring 83.1 (SD 12.1) versus 58 patients in the usual care group scoring 86.1 (SD 7.0) [28].

Emergence Agitation

Xu et al. (2020) reported that the emergence of agitation varied with blood pressure management, with 12 cases at 10% to 20% below baseline, 11 cases at baseline to 10% below, and three cases at baseline to 10% above. A significant difference was observed between the highest group and the other two groups [24]. The full details of the outcome extraction are presented in Table 3.

**Table 3:** Data extraction of the outcomes References: [22-32].

Study ID	Study groups, (n)	Postoperative delirium, no (%)	Delirium score, mean (SD)	Duration of delirium (days), mean (SD)	Hemoglobin change (g/L), mean (SD)	Blood loss (mL), mean (SD)	Length of hospital stay (days), mean (SD)	Other outcomes
Chen 2025 [22]	Tourniquet (n = 157)	30 (19.1)	18 (2.99)	1.67 (0.75)	15.67 (8.23)	Total loss: 553.9 (304)	4.27 (0.86)	61 patients of the tourniquet group and 45 patients of the no tourniquet group experienced several postoperative complications, such as deep vein thrombosis, acute kidney injury, and pneumonia, with no significant difference. 18, 7, and 5 patients experienced hypoactive, hyperactive, and mixed-type delirium, respectively, in the tourniquet group, and 9, 4, and 2 patients in the no tourniquet group
	No tourniquet (n = 156)	15 (9.6)	18.3 (2.24)	1.67 (0.75)	17 (7.48)	Total loss: 600.37 (282.47)	3.50 (0.55)	
Fan 2014 [23]	Restrictive transfusion (n = 94)	20 (21.3)	NA	NA	38.3 (12.6)	Total loss: 481 (176)	8.8 (2.7)	There was no significant difference in pro-inflammatory mediators, such as TNF-α, IL-1b, and IL-6, though IL-8 was significantly higher 4 hours postoperatively in the liberal transfusion group 13 patients the restrictive transfusion group and 18 patients in the liberal transfusion group experienced wound infection, cerebrovascular, cardiac, pulmonary, renal, and urinary tract postoperative complications, with no significant difference
	Liberal transfusion (n = 92)	22 (23.9)	NA	NA	19.3 (13.12)	Total loss: 489 (213)	9.3 (3.9)	
Jing 2022 [25]	Comprehensive care (n = 63)	10 (15.9)	7.1 (2.7)	2.06 (1.03)	NA	NA	11.3 (2.5)	No postoperative complications were reported in both groups
	Conventional care (n = 65)	20 (30.8)	11.2 (3)	3.42 (1.57)	NA	NA	14.2 (2.2)	
Kim 2024 [26]	Blood transfusion (n = 868)	29 (3.34)	NA	NA	NA	Total loss: 938 (881)	NA	In the blood transfusion group fresh frozen plasma transfusion group was associated with a 5.96-fold higher incidence of POD than no transfusion in POD incidence (aOR: 5.96, 95% CI:2.72, 13.04; p < 0.001). Other blood components, like RBC, platelets, and cryoprecipitate, showed no significant difference in POD with the no-transfusion group
	Non-blood transfusion (n = 5869)	138 (2.35)	NA	NA	NA	Total loss: 296 (421)	NA	
Krenk 2012 [27]	Fast-track methodology (n = 225)	0 (0)	NA	NA	NA	NA	2.6 (1.17)	7 patients developed postoperative complications of superficial wound infection, gastric ulcers, and 3 of the 7 patients required reoperation with debridement due to wound complications.
Liang 2021 [28]	Modified Hospital Elder Life Program (n = 81)	2 (2.5)	NA	NA	NA	NA	5.4 (2.3)	In the mHELP group 71 patients scored mean (SD) of 20.8 (4.2) of tMMSE score 1 month postoperatively, and 52 of the usual care group scored 21.1 (4.1) For the ADL score 78 patients of mHELP group scored mean (SD) of 83.1 (12.1), and 58 of the usual care group scored 86.1 (7) after 1 month
	Usual care (n = 59)	3 (5.1)	NA	NA	NA	NA	5.2 (1.7)	
OuYang 2023 [29]	Autologous blood transfusion (n = 401)	8 (2)	NA	NA	NA	Total loss: 266.67 (148.8)	NA	7 patients in the autologous blood transfusion group and 39 in the allogeneic blood transfusion group developed deep vein thrombosis postoperatively and/or required secondary surgery or second hospitalization, with no significant difference, but the lung infection was significantly higher in the allogeneic group, with no correlation between allogeneic transfusion and lung infection after investigation
	Allogeneic blood transfusion (n = 742)	49 (6.6)	NA	NA	NA	Total loss: 333.33 (222.83)	NA	
Petersen 2017 [30]	fast-track methodology with LOS > 4 days (n = 789)	43 (5.45)	NA	NA	NA	NA	10.3 (5.37) in POD patients	No postoperative complications were reported
Ran 2022 [31]	Tourniquet (n = 92)	14 (15.22)	14.24 (1.67)	NA	7.02 (20.56)	Intraoperative loss: 46.67 (15)	NA	There was no difference in postoperative anemia between the 2 groups. There was a significant difference in resting VAS score between 2 groups, with a mean (SD) of 4.43 (0.52) in the tourniquet group and 3.38 (0.69) in the no tourniquet group 1 day postoperatively, 35.87% of tourniquet patients experienced severe pain compared to only 13.04% of patients in the no tourniquet group
	No tourniquet (n = 92)	5 (5.43)	13.82 (1.05)	NA	14.81 (18.4)	Intraoperative loss: 83.33 (22.59)	NA	
Tao 2023 [32]	Active-tDCS (n = 61)	3 (4.9)	27	2	NA	Intraoperative loss: 95 (64.54)	9.83 (2.66)	20 patients of each group developed postoperative adverse events of nausea, vomiting, diarrhea, urinary retention, headache, dizziness, acute left heart failure, and acute respiratory failure with no significant difference. In the active-tDCS group, 1 patient had hypoactive, and 2 had mixed-type delirium; in the sham-tDCS group. 6, 3, and 3 patients showed hypoactive, hyperactive, and mixed-type delirium, respectively
	Sham-tDCS (n = 61)	12 (19.7)	25	2	NA	Intraoperative loss: 108.33 (94.9)	9.67 (3.04)	
Xu 2020 [24]	BP 10% to 20% below the baseline (D) (n = 50)	17 (34)	NA	NA	NA	Total loss: 175 (80)	15 (5)	There was no significant difference in VAS score between the 3 groups, they recorded mean (SD) of 2.2(0.8), 2.4(0.9), and 2.3(0.9), respectively, for the D, M, and H groups. 1 day postoperatively 12 patients in D group, 11 in M group, and 3 in H group developed emergence agitation postoperatively with significant difference between H group and the other 2 groups
	BP from baseline to 10% below the baseline (M) (n = 50)	12 (24)	NA	NA	NA	Total loss: 174 (116)	15 (5)	
	BP from baseline to 10% above the baseline (H) (n = 50)	3 (6)	NA	NA	NA	Total loss: 173 (95)	12 (4)	

Discussion

This systematic review demonstrates that POD in elderly patients undergoing total knee and hip arthroplasty is largely preventable, with incidence varying dramatically based on perioperative management strategies. The range from complete prevention to over one-third of patients affected challenges assumptions that delirium is inevitable and highlights substantial opportunities for quality improvement. Key findings include the following: multimodal interventions consistently outperformed single-component approaches, reflecting delirium's multifactorial pathophysiology; specific modifiable factors, including blood pressure management, tourniquet use, and transfusion practices, showed clear associations with delirium risk; and novel interventions such as transcranial direct current stimulation demonstrated promising neuroprotective effects. These findings indicate that current care variations may contribute to preventable patient harm and that implementing evidence-based, standardized prevention protocols represents a critical quality imperative for this vulnerable population.

Pharmacological interventions for postoperative delirium prevention and management, including antipsychotics, benzodiazepines, alpha-2 agonists, and cholinesterase inhibitors, have been extensively investigated with inconsistent results [33-35]. While these agents may address specific symptoms such as agitation or hallucinations, evidence supporting their preventive efficacy remains limited, and their use is associated with considerable risks, including oversedation, extrapyramidal effects, prolonged cognitive impairment, and increased fall risk in elderly patients [33-36]. Given these limitations and the uncertain risk-benefit profile of pharmacological approaches, non-pharmacological strategies have emerged as safer and potentially more effective alternatives. Non-pharmacological interventions for POD prevention are highly heterogeneous, targeting multiple pathophysiological pathways across the perioperative continuum. These interventions range from systems-level care redesign and surgical technique modifications to anesthetic management strategies, transfusion protocols, environmental modifications, and novel neuromodulatory techniques [37,38].

The non-pharmacological interventions reviewed were highly varied, reflecting diverse approaches to delirium prevention. Some interventions are care-based surgical interventions, such as the fast-track methodology, which represents streamlined perioperative care pathways that emphasize early mobilization, optimized pain management, and accelerated discharge to minimize surgical stress [39]. Comprehensive care protocols are another method that combines cognitive stimulation, sleep optimization, mobilization, and environmental modifications targeting multiple delirium risk factors [40]. Similarly, the Modified Hospital Elder Life Program (mHELP) is a structured care-based intervention integrating cognitive exercises, therapeutic activities, mobility protocols, and sensory adaptations through interdisciplinary teams [41]. Another care-based surgical approach involves tourniquet application, using pneumatic devices to temporarily occlude blood flow during surgery, which reduces intraoperative bleeding but may increase ischemia-reperfusion injury and postoperative pain [22].

While some non-pharmacological interventions are care-based surgical interventions, other interventions focus on anesthetic management strategies during the perioperative period. Blood pressure management represents one such approach, manipulating intraoperative hemodynamic targets ranging from permissive hypotension (10-20% below baseline) to normotensive or slightly hypertensive goals (baseline to 10% above baseline), directly affecting cerebral perfusion and oxygen delivery [24]. Similarly, transfusion strategies constitute another anesthetic consideration, including restrictive approaches that transfuse at hemoglobin thresholds of 7-8 g/dL versus liberal approaches at 9-10 g/dL, as well as choices between autologous transfusion (patient's own pre-donated blood) and allogeneic transfusion with differing immunologic and inflammatory implications [26]. Beyond traditional approaches, tDCS represents a novel neuromodulatory technique that applies weak electrical currents through scalp electrodes to modulate cortical excitability and enhance neuroprotection, with active-tDCS delivering therapeutic stimulation and sham-tDCS serving as a placebo control [32]. Together, these interventions offer multiple approaches to prevent POD by targeting different underlying mechanisms.

The selective association between fresh frozen plasma and increased delirium risk, while other blood components showed no effect, suggests plasma-specific pathophysiological mechanisms. Unlike packed red blood cells, fresh frozen plasma contains bioactive substances, including complement proteins, cytokines, and immunoglobulins, that may trigger neuroinflammatory cascades and disrupt blood-brain barrier integrity [26]. Potential mechanisms include systemic inflammatory activation through complement-mediated responses, immunologic reactions from donor-recipient incompatibilities, alterations in cerebral autoregulation due to rapid volume expansion, and transmission of bioactive lipids or protein aggregates accumulated during storage [26,42].

While cost-effectiveness analyses were not conducted in the included studies, several findings suggest potential economic benefits of evidence-based delirium prevention strategies. Restrictive transfusion strategies showed comparable delirium rates to liberal approaches while reducing healthcare costs and organ dysfunction without compromising outcomes [23,43]. Additionally, integrated care approaches combining psychological, environmental, and pharmacological strategies decrease hospitalization costs [25]. Moreover, Tao et al. (2023) reported lower costs associated with active-tDCS compared to sham-tDCS, although this difference was not statistically significant [32]. Future research incorporating formal cost-effectiveness analyses would provide valuable data to inform resource allocation decisions and support the business case for implementing the results.

This systematic review possesses several methodological strengths. The examination of diverse interventions spanning surgical techniques, anesthetic management, transfusion protocols, and novel therapeutic modalities offers clinicians multiple evidence-based prevention strategies. The substantial patient population across studies and the assessment of various outcomes beyond delirium incidence enable a comprehensive understanding of intervention effects and informed clinical decision-making. Several limitations warrant consideration. The predominance of studies from China and East Asian countries may limit generalizability to other geographic and ethnic populations. Given the nature of the included studies and the methodological heterogeneity in delirium assessment tools, diagnostic criteria, and timing of outcome measurement, direct comparisons or meta-analysis were complicated. Quality assessment revealed concerns about bias in the included studies. Inconsistent reporting of baseline comorbidities introduced uncertainty about population comparability and potential confounding. Small sample sizes across several studies may limit the power to detect meaningful differences. Lack of standardization in delirium subtype classification and severity instruments limited the synthesis of findings. Short follow-up periods precluded assessment of long-term cognitive and functional outcomes.

Future research should address several priorities to advance POD prevention in elderly orthopedic patients. Large-scale, multicenter RCTs with standardized assessment tools and outcome measures are needed to overcome current methodological heterogeneity and enable robust meta-analyses, particularly for promising interventions such as comprehensive care bundles and transcranial direct current stimulation. Studies conducted across diverse geographic regions and ethnic populations would enhance generalizability. Long-term follow-up studies examining cognitive trajectory, functional independence, and quality of life are essential to evaluate the sustained impact of prevention strategies beyond the short-term outcomes reported to date. Additionally, conducting cost-effectiveness analyses of the interventions presented in the included study would enhance decision-making. Finally, studies examining implementation barriers and synergies between multimodal interventions would enhance clinical application.

## Conclusions

This systematic review demonstrates that POD in elderly patients undergoing total knee and hip arthroplasty is preventable, mainly through evidence-based perioperative interventions. Multimodal approaches, including fast-track methodologies, comprehensive care protocols, optimal blood pressure management at or above baseline, judicious transfusion strategies avoiding fresh frozen plasma, and cautious tourniquet use, substantially reduce delirium incidence, severity, and duration. Novel interventions such as transcranial direct current stimulation demonstrate promising neuroprotective effects. The superiority of comprehensive, systems-based approaches over single-component interventions reflects delirium’s multifactorial pathophysiology and underscores the need for integrated prevention frameworks. Implementation of standardized delirium prevention protocols should be prioritized to reduce morbidity, shorten hospital stays, and improve recovery in this vulnerable population. While the evidence provides actionable guidance for immediate practice improvements, critical gaps remain, including the need for standardized assessment tools, multicenter trials across diverse populations, long-term outcome studies, cost-effectiveness analyses, and mechanistic research. Healthcare systems and perioperative teams must translate existing evidence into routine practice to meaningfully reduce the burden of this consequential complication.

## Appendices

Detailed search strategy is given in Table 4.

**Table 4:** Search strategy

Database	Results	Search Query
PubMed	751	(Knee OR Hip) AND (Arthroplasty OR Replacement OR Implantation OR Hemiarthroplasty OR Reconstruction OR Prosthesis OR Endoprosthesis) AND (Delirium* OR Confusion OR "Confusional state" OR "organic mental syndrome" OR "brain failure")
Scopus	561	(ABS((Knee OR Hip) AND (Arthroplasty OR Replacement OR Implantation OR Hemiarthroplasty OR Reconstruction OR Prosthesis OR Endoprosthesis) AND (Delirium* OR Confusion OR "Confusional state" OR "organic mental syndrome" OR "brain failure")) OR TITLE((Knee OR Hip) AND (Arthroplasty OR Replacement OR Implantation OR Hemiarthroplasty OR Reconstruction OR Prosthesis OR Endoprosthesis) AND (Delirium* OR Confusion OR "Confusional state" OR "organic mental syndrome" OR "brain failure")))
Web of Science	742	TS=((Knee OR Hip) AND (Arthroplasty OR Replacement OR Implantation OR Hemiarthroplasty OR Reconstruction OR Prosthesis OR Endoprosthesis) AND (Delirium* OR Confusion OR "Confusional state" OR "organic mental syndrome" OR "brain failure"))
Cochrane Library	391	((Knee OR Hip) AND (Arthroplasty OR Replacement OR Implantation OR Hemiarthroplasty OR Reconstruction OR Prosthesis OR Endoprosthesis) AND (Delirium* OR Confusion OR "Confusional state" OR "organic mental syndrome" OR "brain failure")):ti,ab,kw
Embase	788	(knee:ab,ti OR hip:ab,ti) AND (arthroplasty:ab,ti OR replacement:ab,ti OR implantation:ab,ti OR hemiarthroplasty:ab,ti OR reconstruction:ab,ti OR prosthesis:ab,ti OR endoprosthesis:ab,ti) AND (delirium*:ab,ti OR confusion:ab,ti OR 'confusional state':ab,ti OR 'organic mental syndrome':ab,ti OR 'brain failure':ab,ti)
